# Enhanced Milieu Teaching in resource-constrained settings: Stakeholder-informed adaptation guidelines

**DOI:** 10.4102/sajcd.v71i1.989

**Published:** 2024-02-21

**Authors:** Chevonne D. du Plessis, Lauren H. Hampton, Michal Harty

**Affiliations:** 1 Department of Health and Rehabilitation Sciences, Faculty of Health Sciences, University of Cape Town, Cape Town, South Africa; 2 Department of Special Education, The University of Texas at Austin, Austin, United States; 3 Centre for Autism Research in Africa, Faculty of Health Sciences, University of Cape Town, Cape Town, South Africa

**Keywords:** adaptations, contextual fit, developmental disabilities, Enhanced Milieu Teaching, implementation science, naturalistic developmental behavioural interventions, resource-constrained settings, stakeholder perceptions

## Abstract

**Background:**

Enhanced Milieu Teaching (EMT) is an evidence-based naturalistic developmental behavioural intervention (NDBI) for children with developmental disabilities. Little is known about the EMT’s fit or what adaptations might be needed to improve its applicability within a resource-constrained setting.

**Objectives:**

To explore stakeholders’ perceptions of the contextual fit of EMT for young children with developmental disabilities in a resource-constrained context and to identify adaptations to improve EMT’s contextual fit.

**Method:**

We conducted a descriptive qualitative study using semi-structured interviews and focus groups. Participants included 5 speech and language therapists and 11 caregivers of children with developmental disabilities who speak English and Afrikaans or isiXhosa. Using thematic analysis, data were coded into 10 subthemes and grouped according to the Adaptome framework components.

**Results:**

Overall, stakeholders view EMT as an appropriate intervention in the South African context. They indicated that certain intervention components may need to be modified. Specifically, clinicians may need to adapt intervention materials and activities to be sensitive to families’ available resources, preferred activity routines and priorities. From these data, we provide guidelines to improve the fit of EMT in South Africa.

**Conclusion:**

Enhanced Milieu Teaching is an appropriate intervention in the South African context, although some adaptations can enhance its fit.

**Contribution:**

This article highlights the importance of engaging with stakeholders to determine the fit of NDBIs, like EMT, as they are implemented in new contexts. Based on these insights, stakeholder-informed adaptation guidelines are provided for improving the contextual fit of EMT in resource-constrained settings.

## Introduction

The majority of children with developmental disabilities and their families do not have sufficient access to evidence-based early intervention services in South Africa (Samuels et al., 2012). Collaborating with caregivers is the foundation of early intervention services and many evidence-based interventions have a component of caregiver coaching (Makombe et al., 2019; Movahedazarhouligh, 2021; Seruya et al., 2022). The most promising category of caregiver-mediated interventions is naturalistic developmental behavioural interventions (NDBIs) (Sandbank et al., 2020). In high-income countries (HICs), NDBIs are considered best practice for children with autism (Sandbank et al., 2020), and some of these interventions are being used effectively for children with other developmental disabilities (Kaiser & Hampton, 2016). Naturalistic developmental behavioural interventions are a set of child-centred interventions that use natural and behavioural contingencies to teach developmentally appropriate skills in natural settings (Schreibman et al., 2015).

Naturalistic developmental behavioural interventions are considered to be effective because they are based upon the principles that children learn best when experiences are developmentally appropriate and when they are allowed to be active participants in the environment (Schreibman et al., 2015). These authors add that they are implemented in naturally occurring environments and incorporate a variety of behavioural elements to teach skills to facilitate language growth and development. Furthermore, caregivers and other professionals have been successfully taught to implement NDBIs with sufficient training (Movahedazarhouligh, 2021). This means that over time, these caregiver-mediated interventions allow children to benefit from increased intervention dosage compared to traditional clinician-led intervention approaches as trained caregivers can continue to use the skills in the home context without the need for a clinician to be present (Kaiser & Roberts, 2011). As NDBIs allow for a systematic transfer of skills from highly skilled professionals to caregivers (and other healthcare workers or educators), they hold promise as an effective intervention approach in resource-constrained contexts such as South Africa.

There is increased interest in applying NDBIs in low- and middle-income country (LMIC) contexts, because of the flexibility in how they can be implemented (Schlebusch et al., 2020). While the effectiveness of these interventions has been demonstrated in HICs, the majority of NDBIs have not been systemically implemented with culturally and linguistically diverse children and families who live in LMIC contexts (Schlebusch et al., 2020). However, one of the most frequently cited barriers in the implementation process is a lack of ‘fit’ between the intervention, which may have been developed elsewhere, and the current practice setting. This has also been called ‘appropriateness’ (Proctor et al., 2011). The field of implementation science provides frameworks that allow us to document the process of implementing existing interventions in novel settings which differ from the ones in which they were developed (Bauer et al., 2015; Proctor et al., 2009, 2011; Theobald et al., 2018).

Within the implementation science literature, an intervention is said to possess good contextual fit when a broad range of stakeholders identify the intervention as acceptable, doable, effective and sustainable. In other words, contextual fit is defined by those who will be implementing, supporting and receiving the intervention (Damschroder et al., 2009). It is important that *fit*, as a construct, should take into account various stakeholder perspectives at different access points in the service delivery systems (Damschroder et al., 2009). Favourable stakeholder views on the contextual fit (applicability) of an existing intervention within a novel setting are essential for the initial uptake (adoption), as well as the long-term sustainability of the intervention.

Maximising the *fit* of an intervention within a new setting is especially important where the current setting and target population differ from the original setting and trial population. There are many documented reasons why an intervention may not fit optimally in a new context. These differences can include client factors such as age, race, ethnicity, culture and language; contextual factors such as intervention accessibility, dosage and intensity, as well as systemic issues such as staffing and resource limitations (Chambers & Norton, 2016). One mechanism to improve the fit of an intervention within a specific context is to adapt certain components of the intervention. The challenge is to adapt the components that will enhance fit while still retaining the components that underpin the effectiveness of the original intervention. A distinction therefore needs to be made between the core components and the adaptable periphery of an intervention. The core components of an intervention constitute the essential components that should not require adaptation. The adaptable periphery of an intervention encompasses all the structures, systems and adaptable elements that could be modified across diverse service settings and service delivery levels (Damschroder et al., 2009). Our study uses the Adaptome framework of Chambers and Norton (2016) who refer to four peripheral intervention adaptations, namely, service setting adaptations, target audience adaptations, mode of delivery adaptations and cultural adaptations.

Preliminary data relating to intervention adaptations of caregiver coaching interventions are beginning to emerge from LMIC settings. Examples recently documented include mode of delivery adaptations, such as the move from in-person caregiver coaching sessions to telehealth sessions during the coronavirus disease 2019 (COVID-19) pandemic (Franz et al., 2022); cultural adaptations, such as adapting the concept of relational communication therapy to be taught to caregivers in a Tanzanian orphanage (Schütte, 2016); target audience adaptations, such as changes to the text and manuals of the World Health Organizations Caregiver Skills Training Well-Being Module (Schlebusch et al., 2022) and service setting adaptations, such as adapting delivery of caregiver coaching intervention by non-specialist Early Childhood Development practitioners (Dunn Davison et al., 2021; Kasari et al., 2014; Makombe et al., 2019). For most interventions developed in HICs, adaptations will need to be made for the intervention to work in an LMIC context. Furthermore, appropriate adaptations play an important part in ensuring that the intervention will continue to be implemented and have the desired outcomes.

South Africa, a middle-income country, remains as one of the most unequal societies in the world. Despite over 20 years of democracy, the majority of South Africans continue to face challenges such as weak healthcare systems, poor infrastructure and constrained resources. As a consequence of this inequality, Popich, Louw and Eloff (2007) indicate that 84% of South African caregivers are unable to access regular intervention services for their child with a developmental disability. There is increasing interest in utilising NDBIs in the South African context to address some of these constraints. Enhanced Milieu Teaching is one example of an NDBI currently being implemented in South Africa (Hampton et al., 2019). Enhanced Milieu Teaching focusses on increasing a child’s communication abilities within the context of social interaction (Kaiser et al., 2013). It is a hybrid intervention consisting of six main strategies: environmental arrangement, responsive interaction, target-level language, expansions, time delays and milieu prompting (Kaiser & Hampton, 2016). It has been found to be effective for children with cognitive delays (Kaiser et al., 2013), children with specific language impairments (Roberts & Kaiser, 2012; Roberts et al., 2014), children with autism spectrum disorder (Dunn Davison et al., 2021; Kasari et al., 2014) and children with Down syndrome (Kaiser & Roberts, 2013; Wright et al., 2013). More than 50 empirical studies have provided evidence for the efficacy and effectiveness of EMT. Furthermore, EMT has been effectively implemented in resource-constrained contexts in HICs (Hatcher & Page, 2020). Enhanced Milieu Teaching is also one of the few NDBIs that has been implemented in languages other than English (Peredo et al., 2018, 2022). An earlier exploratory study by Hampton et al. (2019) demonstrated that the expressive language development of English-speaking children with autism improved after a period of EMT intervention provided within a South African special school setting.

Despite this exploratory study by Hampton et al. (2019) which demonstrated that EMT is effective as an NDBI in a controlled setting, little is known about the adaptations that might be needed to improve the fit of EMT within a resource-constrained setting. According to implementation science literature, determining contextual fit is best done by involving stakeholders. In this study we focused on two stakeholder groups: those who conducted the intervention (i.e. speech-language therapists [SLTs]) and those who were the recipients of the intervention (i.e. caregivers of children with developmental disabilities). These two groups of stakeholders are directly involved in the implementation process and therefore are the most likely to be able to provide information relevant to this study. Therefore, the aim of this study was to identify a group of SLTs and caregivers’ perceptions of the contextual fit of EMT for young children with developmental disabilities in a resource-constrained context. These stakeholder-informed adaptation guidelines can be used to improve EMT’s fit in a resource-constrained context and in so doing increase appropriate NDBI options available in LMICs.

## Methods

### Design and objectives

The objectives of the study were to: (1) determine caregiver and SLT perceptions regarding the contextual fit of EMT components, namely the intervention targets, materials and strategies within a culturally and linguistically diverse South African context and (2) identify adaptations needed to improve EMT’s fit in South Africa, based on these perceptions. A descriptive, qualitative design was used in this study as it allowed the researcher to achieve detailed descriptions from various stakeholders (Doyle et al., 2020). Data were collected using semi-structured interviews with 5 SLTs and two focus groups with a total of 11 caregivers. The data analysis process was informed by intervention adaptation literature from the field of implementation science, specifically the Adaptome framework.

### Participant selection and recruitment

The study specifically recruited bilingual caregivers and therapists to ensure that the multilingual nature of the South African context was considered. As the study was situated in the Western Cape, we focused on bi- or multilingual caregivers and therapists who spoke English, isiXhosa and/or Afrikaans, which are the most dominant languages of the province. Caregivers were recruited from two schools catering to children with developmental disabilities in the Western Cape Province.

Caregivers were asked to participate in the study if they: (1) had a child (aged 5–10 years) with a developmental disorder diagnosis placed at the target schools, (2) conversed with their child daily in either Afrikaans or isiXhosa and (3) were fluent in English. Speech-language therapists were asked to participate if they: (1) had at least 3 years’ experience in working with multilingual children with developmental disabilities, (2) identified as bi/multilingual and (3) had English, Afrikaans and/or isiXhosa as a home language. The SLTs were recruited via snowball sampling.

### Participant description

#### Speech-language therapists

Seven SLTs were initially contacted and five SLTs provided written consent to participate in the study. The mean age of SLT participants was 32 years. Speech-language therapists were equally represented on key sociodemographic characteristics (see Table 1). All SLTs were female.

**TABLE 1:** Speech-language therapist’s interview participant demographics (*N* = 5).

Demographics	Number of SLTs
**Age range (years)**	
21–30	2
31–40	3
**Languages that SLT provides intervention in**	
Afrikaans/English	2
IsiXhosa/English	3
**Years of experience**	
3–5	3
6+	2
**Service sector employment**	
Public	2
Private	3

SLT, Speech-language therapist.

#### Caregivers

A total of 40 consent letters were sent out to families. Thirteen caregivers provided consent and 11 took part in the focus groups as 2 caregivers were unable to attend on the day the focus group was held. Table 2 provides an overview of the caregiver’s sociodemographic characteristics. The mean age of the children was 5 years and the mean age of caregivers was 38 years. The children’s diagnoses included autism spectrum disorder (ASD), global developmental delay (GDD), Williams syndrome and Prader-Willi syndrome.

**TABLE 2:** Parent focus group participant demographics (*N* = 11).

Demographics	Number of caregivers
**Age range (years)**	
21–30	1
31–40	7
41–50	2
51+	1
**Gender**	
Male	4
Female	7
**Languages spoken in the home or other environment**	
Afrikaans/English	8
isiXhosa/English	3
**Age of child (years)**	
3–6	7
7+	4

### Data collection methods

Both the focus group and semi-structured interview participants viewed video material of EMT being implemented with a young child in a first-world setting (the United States) obtained from the developer of EMT, as well as video material within a local (South African) context. Both videos demonstrated EMT being implemented with an English-speaking child within an everyday home routine (mealtime). Interview and focus group participants were then asked a series of questions to establish their perceptions of the fit of EMT within the culturally and linguistically diverse South African context. The interview guide was developed from key adaptation concepts in implementation science, namely appropriateness and acceptability (Proctor et al., 2011), and included open-ended questions about:

- the appropriateness of EMT (the benefits and challenges of implementing/receiving this intervention; the relevance of these intervention goals/activities to your child/children on your caseload),
- the acceptability of EMT (feelings towards this intervention; willingness to recommend it to a friend/family),
- adaptations needed for EMT (changes needed to the materials/goals to make the intervention more suited to your child/children on your caseload).

### Semi-structured interviews

Three of the semi-structured interviews were conducted face-to-face in a setting easily accessible to the participants. The remaining two interviews were conducted via Skype. The interviews lasted between 60 and 90 min each and took place in a quiet location after work hours to minimise distractions (Krueger & Casey, 2009). The interviews took place in English as all the SLTs were fluent in English.

### Focus group discussions

The two focus groups with caregivers were interviewed at a venue easily accessible to the participants and lasted between 60 and 90 min. Two female researchers were present for the duration of each of the focus groups, one of whom had extensive experience in implementing EMT and interviewing caregivers of children with disabilities. The focus group questions were posted in English; however, a male multilingual (Afrikaans, English, IsiXhosa) research assistant was present at both discussions and participants were encouraged to converse in the language of their choice throughout the focus group. The role of the research assistant was to translate where needed to ensure that all group participants were able to contribute equally to the discussion. Participants were reimbursed for travel costs to the venue.

Both the semi-structured interviews and focus group discussions were audio-recorded, transcribed verbatim and emailed to the participants within seven working days for member checking (Cresswell et al., 2003). None of the participants indicated they required changes to the transcripts.

## Data analysis

A thematic analysis was conducted using Dedoose (Dedoose Version 9.0.86, 2023). Data were analysed using the steps outlined in Neuman (2014). Data were initially organised into meaningful units or codes. Next a coding rubric was developed, and all the codes were grouped into subthemes. Data were analysed by two members of the researcher team and initial coding discrepancies were resolved using consensus methodology among these two researchers. The 10 subthemes were then grouped into 5 themes according to the adaptation components in the Adaptome framework (Chambers & Norton, 2016). This revised coding rubric and 25% of the coded transcripts were sent to a qualified SLT for independent analysis to ensure credibility and dependability of the findings. Once again, discrepancies were resolved among the researchers using consensus methodology.

## Findings

Caregivers and clinician’s responses are reported according to five themes: a ‘core component’ theme (which we used to indicate the overall fit of the core elements of EMT) and four adaptation themes, namely service setting adaptations, target audience adaptations, mode of delivery adaptations and cultural adaptations, as described in the Adaptome framework (Chambers & Norton, 2016).

### Enhanced Milieu Teaching core component fit

Core component fit encompasses stakeholders’ perceptions about the overall fit of EMT within their context. Stakeholders expressed a positive attitude towards the EMT components demonstrated in the video material. They indicated that the EMT could work ‘very well’ in the South African context. Caregiver 1 commented that they ‘like[*d*] the sessions’ as EMT ‘fits in’ well with what they already know about communication intervention. In addition, clinicians liked the naturalistic approach of EMT, as well as its potential to ‘empower caregivers’. Clinician 5 said:

‘… so, it would be very easy to carry on and because I know that I do have families that are really willing and willing to do things at home and then also, thinking about if we could, let’s say we need so many sessions, and then if the parent could carry on at home, like that’s really, I’d say cost effective and time effective …’

Clinician 2 commented that:

‘… everybody in this context would benefit from it, it would just need tweaking to be contextually appropriate.’

It is evident that stakeholders can see the value of implementing naturalistic interventions, such as EMT, in their context. Additional perceptions regarding the nature of these ‘tweaks’ are presented in the remaining adaptation components of the Adaptome framework.

### Service setting adaptations

This theme highlights challenges relating to providing EMT within existing service settings. Three subthemes were identified, namely, physical intervention settings, accessing consistent intervention partners and training requirements.

The physical intervention setting was mentioned by clinicians from both the public and private sectors who raised concerns regarding ‘large caseloads’ and limited session duration and/or frequency within the clinic setting as barriers to implementing EMT. Clinicians in the public sector noted difficulty in caregivers accessing clinic-based services and, vice versa, for them to access home environments because of remote locations, resulting in them seeing children inconsistently. Clinician 2 highlighted the impact of this inconsistency on intervention outcomes:

‘Because that’s also the reality of this context … that you don’t always have access to the child as regularly as you would like so that the carryover isn’t the way that you would like for it to be.’

In addition, clinicians stated that accessing consistent communication partners is difficult because different caregivers bring the child to the clinic for intervention. This can make it difficult to train caregivers. Clinician 5 said:

‘We have families coming and today is the grandmother bringing the child, and the next time it’s the neighbour bringing the child, then it’s that one bringing the child so it’s very difficult to try and train a parent how to be doing these things at home.’

Caregivers acknowledged the challenges of making themselves available but indicated that they were willing to work together to overcome this challenge. Caregiver 4 made this suggestion:

‘I mean, one of us would be available … at all times. It might not be both of us at the same time, but we could take turns.’

It is clear that caregivers are willing to be trained but constraints exist for how to do this practically.

Both stakeholder groups indicated a willingness to learn how to implement EMT. Caregivers indicated that they were ‘more than prepared’ to learn EMT. Caregiver 3 pointed out:

‘Any parents would want to do it; anything to make things easier for [*the child*] so they feel more comfortable to say to you: “I need this.”’

Clinician 5 summed up her feelings about learning the intervention:

‘Your caseload is quite large so, if you could [*decide*], “okay, we’re going to do EMT with this family and it’s going to be for so many weeks … it would be quite cool.”’

However, despite their willingness to learn EMT, caregivers and clinicians raised some concerns. Caregivers indicated that they would require specific training material to help them decide ‘when can I, and when can I not’ when trying to implement EMT with their child at home. Caregiver 4 stated:

‘We try to bring in what was shown there [*in the EMT video*] every day, but I think if we just know which moments are right, that would help a lot.’

Caregivers said they found implementing intervention at home to be ‘difficult’ and Caregiver 3 shared that they often wondered:

‘Is what I’m seeing a good time [*to try this*] or a bad time?’

Despite a clear need and appetite for EMT training, both stakeholder groups raised concerns about the logistical constraints of face-to face training opportunities, such as timing and location, which may make it difficult to meet training requirements. Clinician 5 asked for additional information relating to training logistics:

‘I would definitely want to know…what is the average time that you would need … yourself to be trained, and then the caregiver to be trained … because [*time is*] also a big thing in government sector.’

Caregiver 3 proposed an online ‘platform’ as a potential strategy to overcome these challenges:

‘… I think it’s also important to do [*EMT training*] electronically… that there is a system that allows people to do the best they can according to their times.’

### Mode of delivery adaptations

Stakeholders highlighted elements of the actual EMT intervention sessions which may need to be adapted. Two subthemes were identified, namely, creating natural opportunities to communicate and choosing appropriate intervention activities and materials.

Clinicians and caregivers highlighted the value of creating an environment where interaction happens naturally and the ‘child interacts very freely’. Clinician 4 pointed out that:

‘The more they [*parents*] can create a natural environment and communicate with their children and expand on their children’s utterances, the more the child will benefit.’

This sentiment is echoed by Caregiver 3 who stated:

‘It’s so exciting to know that I can learn to give him the control in certain things, you know. To put the scenario in play and then [*wait for*] him to start doing something.’

Clinicians identified the importance of using individual child interests to maximise child engagement. Clinician 5 said:

‘I’d always ask the parent … and sort of try and work more towards that so … activities we do are more like feeding and playing with maybe a baby doll and pretending to cook food. Things that are a bit more appropriate than just playing with maybe just cars.’

Caregivers echoed the importance of identifying their child’s level of play and their interest in toys, before deciding on what activities should be used as the basis for teaching language. Caregiver 8 highlighted that their child’s interest is in real objects not toys:

‘Give him a real hammer, give him a spade. He’s not interested to play … I can tell you he won’t sit there and play with us.’

Both stakeholder groups identified the need to choose relevant intervention activities and materials for individual families and their context. Clinicians from both the private and public sectors noted from the videos shared that the intervention is play-based. Clinicians within the private sector felt that most families on their caseloads would have access to the types of toy sets used within EMT in their home environments, and stated they frequently used play-based activities in therapy. Clinician 1 shared that she uses toys in her sessions:

‘Look, we are play-based, we use toys in what we do … our children know this. I think they identify with this [*approach*].’

However, clinicians in the public sector tended to favour everyday activities above play-based activities, as they indicated that children’s exposure to play and toys varied significantly. Clinician 5 shared their caution regarding the use of toys in intervention:

‘There was a lot of things that I was thinking of that might not work so well, especially if we think about our population and sort of the things that our population has at home. [*In the video*] they were using all these fancy toys, and this is something not really that our population would have at home.’

Caregivers reported that their children were not always interested in toys or games and they prefer to teach their child within everyday activities or familiar family routines. Caregiver 7 commented:

‘When we are at home, then I will take the macaroni and the strainer, and then [*when*] we start doing it, he will start being interested in it.’

Caregivers therefore highlighted the importance of choosing intervention activities and materials that are appropriate to a child’s interest and developmental abilities.

### Target audience adaptations

This theme focused on considerations that may need to be made for families of children with developmental disabilities. A total of three subthemes were identified, namely, caregiver empowerment and advocacy, family priorities and routines, and families’ access to resources.

Enhanced Milieu Teaching can be implemented by both clinicians and caregivers, and this flexibility is part of the appeal of implementing EMT in the South African context. Most participants saw EMT as an advocacy and empowerment tool for caregivers. Clinician 5 stated:

‘I think it might be quite a way to empower parents.’

Caregivers highlighted the fact that they spend more time than the clinicians with their child and that they would therefore be good implementation partners. Caregiver 4 explained it as follows:

‘This EMT programme needs to empower and upskill you, because [*if*] you learn the right ways … you don’t have 1 hour a week, you have 50.’

Caregiver 2 stated that:

‘It’s a bit of a light that’s gone on for me, you know. I think we could do much more. Give independence to a child when you wait for a cue, or when he does something …’

However, as mentioned in the previous theme, caregivers would need to be trained before they are able to implement EMT with their children.

Both stakeholder groups cautioned that family priorities and routines differ and indicated that these priorities and routines would need to be considered when deciding if and when to implement EMT with a family. For instance, not all caregivers indicated that they are concerned about their child’s communication abilities at that point in time. Caregiver 8 indicated that their immediate priority was the safety of their child and said their concern is ‘not really … a speech problem …’ but rather that their child does not recognise danger and has ‘no fear’. Caregiver 8 shared that their child is ‘all on his own with us’ and ‘doesn’t really play with other children’ so in their view ‘he doesn’t really need to communicate …’

It is clear that, at least for some families, current caregiver priorities do not align with the espoused goals of EMT intervention.

Both groups of stakeholders also mentioned aspects such as financial and time constraints which need to be considered when deciding to implement EMT with a family. In terms of time constraints, Clinician 5 summarised the complexities in this way:

‘Like if it’s a mum, who is a single mother and she has 4 kids … it’s not something that’s as important because they need to focus about getting food on the table. There are some factors, but I think it really is just something that just depends on the family, and it depends on the parent.’

Caregiver 9 indicated the challenges of working long hours and states how this impacts on the amount of time the family has to complete family activities together.

‘I come late home. His mother also is coming [*home late*] from work. She is very tired. To focus a lot on [*child’s name*] … it [*is a*] little bit difficult for us.’

Caregiver 7 echoed this concern and stated:

‘Because you would only be able to give him, most probably, even if it’s in that normal household setup, probably an hour before bath time or something like that, because after bath time it’s finished.’

Finally, families’ access to resources was discussed frequently among both the caregivers and clinicians. Clinicians and caregivers were aware of the potential mismatch between the resources available in the therapy setting and what caregivers may have access to at home. As Caregiver 1 said:

‘Money is an issue. It’s a reality, you know, not everyone can afford this.’

This was echoed by Clinician 3 who stated:

‘… and maybe they [*caregivers*] want to use the same types of things [*toys*] but … they’re not always in a financial position to afford that, especially within the public sector.’

Overall, caregiver empowerment was seen as a strength of this approach. However, resource constraints because of socioeconomic realities, as well as family routines and preferences, were foregrounded as potential areas for adaptations to consider when implementing EMT. It is clear that clinicians need to work together with caregivers to tailor the intervention to each family.

### Cultural adaptations

This theme highlighted aspects of multiculturalism and multilingualism which need to be considered when implementing EMT in the South African context. A total of two subthemes were identified, namely multilingual intervention targets and diverse parenting beliefs and practices.

The majority of the caregivers acknowledged the multilingual nature of their communication with their child. Many families in South Africa are bilingual or multilingual. As a result, EMT needs to consider the family’s preferences towards which language(s) should be targeted during intervention, as well as the specific language targets which form the focus of the intervention. Caregiver 8 explained the code switching that their child is exposed to even though the family predominantly speaks Afrikaans at home. They said:

‘I speak to [*child’s name*] normally in Afrikaans. Some words it’s just, like, Afrikaans, and … But most of his, the three-letter words, that mostly is English. Even here, there, is Afrikaans, or Pedi, or like that.’

Caregivers are also concerned about how to adapt language input in the home because of their child’s developmental disability. Caregiver 9 explained how they stopped speaking in their home language to their son because of his communication impairment:

‘We used to communicate to [*him in*] his mother’s language, Xhosa, and [*in*] English. But [*to use*] my home language, Lingala, around [*him*] … because I know his situation … I … [*reminded*] myself [*not to use*] too much [*sic*] languages.’

Clinician 2 pointed out how she ‘needs to be mindful’ about language exposure.

‘… I try very hard to … get as much information as I can out of the caregiver in terms of the language exposure, what are all the languages they are exposed to? Okay, what are all the different dialectical differences because in Xhosa even there’s different dialects …’

In addition, clinicians highlighted the challenges of coaching caregivers to work with their child when therapists are not fluent in the family’s home language. Clinician 5 pointed out:

‘It’s so much easier to show a mum something physical like you’re trying to put up a child’s hands, but now you have to explain to her how to communicate with her child and it’s already a different language.’

It is also important to consider that specific parenting beliefs and practices are culturally informed, and these parenting beliefs may shape the nature and frequency of interactions between the child and other adult members of the family. Clinician 2 shared her experience about the impact of parenting styles on intervention carryover into the home:

‘The culture practice at home in terms of communicating with the kids … is very important as well. It is more of a “seen than be heard” situation then there is no integration [*at home*].’

Some caregivers also clearly identify with a more directive style of interaction, which may make implementing certain EMT strategies (such as responsiveness) more challenging. Caregiver 7 explained their views as follows:

‘For me, it’s to follow a command, because if I send him to do something he still doesn’t. That is what I want. That will make me happy.’

Overall, defining what culturally appropriate communication means for a family is a necessary step to identify appropriate EMT strategies to employ during intervention.

## Discussion

In this study we obtained perceptions of the contextual fit of EMT for young children with developmental disabilities in a resource-constrained context from two stakeholder groups. Caregivers and SLTs were asked about the appropriateness of EMT and what aspects might need to be adapted to maximise the fit of the intervention in their context. Informed by the implementation science literature, we used the Adaptome framework (Chambers & Norton, 2016) to categorise these perceptions into the broad adaptation components, namely, service setting adaptations, mode of intervention delivery adaptations, target audience adaptations and cultural adaptations. We discuss key findings from the data and present four specific adaptations that would be needed to improve EMT’s fit in a resource-constrained context and in so doing increase appropriate NDBI options available in LMICs.

Both stakeholder groups perceive EMT as applicable in the South African context. They indicate that EMT is appealing because it can be implemented using everyday routines in the home context. This finding is congruent with previous South African service delivery research where caregivers of children with autism preferred home-based early intervention because of the challenges in accessing clinic-based services (Guler et al., 2018; Moolman-Smook et al., 2008). Both groups of stakeholders indicated that they wanted to receive EMT training, which also indicates that they can see the value of EMT in their context. However, the time allocation, cost and format of the training need careful consideration. The expense of completing training has been raised by other South African researchers as a barrier to implementing new interventions in LMICs (Dawson-Squibb et al., 2022).

Both groups of stakeholders agreed that there is a need to consider families and work closely with caregivers. The necessity for caregiver training and empowerment is key, especially in contexts where there is a cultural and linguistic mismatch between professionals and families. While training caregivers is not a new concept in the task sharing literature (Rahman et al., 2016), collaboration needs to move beyond caregiver training and empowerment and towards acknowledging a family’s role as ‘cultural brokers’. Cultural brokerage is the intentional use of culturally competent strategies to bridge or mediate between the patient’s culture and the biomedical healthcare system (Hodge et al., 2016; Kinnaird, 2007; Pang et al., 2019). Caregivers can act as a bridge between their children and service providers. Caregivers can ‘educate’ professionals as to what is appropriate and feasible within their setting by sharing information about their parenting beliefs and approaches to caregiver–child interactions. Peredo et al. (2018, 2022) reported that caregivers’ help shaped both the linguistic and cultural adaptations that were required to support EMT implementation with Spanish-speaking caregivers from low-resource settings. Caregivers guided clinicians to individualise intervention target words, based on the dialect of Spanish that was spoken in their home. In addition, the intervention strategy of giving children choices, which is culturally inappropriate in Spanish-speaking families, was omitted from the intervention after consultation with caregivers.

Both groups of stakeholders agreed that there is a need to consider families individually and work closely with them to identify appropriate routines that they can use to practice with their child. Caregivers stressed the importance that the SLTs take the time to understand the child’s current communication abilities and levels of engagement (for example, a preference for real objects embedded in everyday activities rather than toys embedded in play activities). Peredo et al. (2018) provide some examples of the adaptations they made when teaching EMT to Spanish-speaking caregivers such as including everyday activities such as mealtimes as an alternative activity to play-based interactions. They also offered more directed activities such as story book reading and storytelling for those caregivers who were less comfortable engaging in free play with their child. These adaptations thus indicate that it is possible to implement intervention while being sensitive to cultural factors influencing a family, as well as individual child preferences (real objects versus toys). Several researchers have interviewed caregivers and children with intellectual disabilities about participation in daily activities. The results of these studies could provide an exemplar list of daily activities which might be appropriate in a South African context (Balton et al., 2019; Huus et al., 2021; Ramseur et al., 2019; Schlebusch et al., 2016, 2020).

Finally, stakeholders are concerned about the impact of resource constraints on their ability to implement EMT in their context. Limited funds to purchase intervention materials, such as toys, were raised by both clinicians and caregivers. Clinicians and caregivers were aware of the potential mismatch between the toys available in the intervention setting and what caregivers may have access to at home. The other resource mentioned by the stakeholders was time, specifically in relation to implementing EMT in the home. Once again this is a common barrier reported in the literature (Makombe et al., 2019). In this study, caregivers’ views on the aspect of time varied widely. Some caregivers noted that they had limited time to implement EMT in the home context, while others acknowledge that they had more time than the clinicians to interact with their child and would welcome the opportunity to implement EMT at home.

From these key findings we extrapolated four considerations that would improve the fit of EMT in the South African context. They are:

- *Implement EMT in daily activities in the home context wherever possible*. Implementing EMT in everyday routines (such as mealtimes) may be a viable alternative to play-based EMT intervention, where caregiver–child play may not be seen as culturally appropriate, or access to play material is limited.
- *Develop culturally appropriate training materials, accessible to caregivers from diverse backgrounds, to empower caregivers as collaborative intervention partners*. Authors such as Franz et al. (2022) and Schlebusch et al. (2022) can be consulted as they have documented the changes made to training materials before implementing their interventions in the South African context.
- *Engage caregivers as cultural brokers when implementing EMT*. In South Africa, most SLTs do not speak the home language of the families they are serving. Therefore it is essential to recruit the family as cultural brokers. Training caregivers to use EMT within the home context may increase the likelihood of generalisation and may mitigate some of the access issues many families face. However, clinicians need to go one step further and learn from caregivers about the parenting beliefs and practices that are important to them if intervention is to be acceptable and appropriate to families.
- *Address obstacles to current training opportunities, by using an online/blended training approach which incorporates web-based technology, such as Skype or Zoom*. Caregivers in this study indicated the need to have trainings and training material available in an online format. Notwithstanding the challenges around technology access in low- and middle-income contexts (Kumm et al., 2022), some pioneering work using telehealth to implement NDBIs is being conducted in South Africa (Franz et al., 2021).

## Study strength and limitations

This study extends the evidence of stakeholder perceptions of implementing NDBIs in LMICs. Perceptions of a diverse group of clinicians and caregivers were included whereas most existing empirical studies to date have focused on collecting data from a single group of stakeholders.

There are several limitations to the study. South Africa is a culturally and linguistically diverse country, and these findings may not be representative of the views of caregivers with differing cultural and linguistic beliefs. In addition, we did not source administrative and managerial stakeholders’ perceptions about the applicability of EMT. This element is important for future research as these stakeholders would need to support staff to implement the above adaptations. Organisational stakeholders’ buy-in is crucial for the adoption and sustained implementation of NDBIs within local contexts. Finally, we acknowledge that stakeholder perceptions are based on video recordings of the intervention rather than on actual experiences of receiving or delivering the intervention.

## Conclusion

This study explored caregiver and clinician perspectives on the fit of EMT in South Africa based on stakeholder’s perceptions of EMT after watching two video recordings of EMT being implemented with a young child. Both stakeholder groups perceived EMT to be applicable in South Africa. Information gathered from stakeholders indicated that certain intervention components need to align better with elements of a family’s social and cultural context to improve the overall fit of naturalistic interventions in a culturally and linguistically diverse low-resource setting. Based on these stakeholder insights, guidelines for adaptation were provided for improving the contextual fit of EMT in resource-constrained settings like South Africa. As NDBIs like EMT are being implemented in low-resource contexts, stakeholder perceptions about necessary adaptations contribute to ensuring NDBIs are viewed as relevant in low-resource contexts.
